# Supplementary material for: Are carbon emissions trading and green financial instruments synergistic? -Comprehensive quantitative research based on content analysis
Source: PLoS One. 2024 Mar 7;19(3):e0298601. doi: 10.1371/journal.pone.0298601 (PMC10919601; doi:10.1371/journal.pone.0298601)
Supplement: S1 Data — (ZIP) [file pone.0298601.s001.zip › Data/地级市Z_F工作报告.zip!/─┌├╔╣┼/║⌠║═║╞╠╪╩╨/└┤╫╘╣1⁄2╓┌║┼í╛┬φ┐╦╩2╛▌═°í┐_2015.docx]

各位代表：

现在，我代表国务院，向大会报告政府工作，请予审议，并请全国政协各位委员提出意见。

一、2014年工作回顾

过去一年，我国发展面临的国际国内环境复杂严峻。全球经济复苏艰难曲折，主要经济体走势分化。国内经济下行压力持续加大，多重困难和挑战相互交织。在以习近平同志为核心的党中央坚强领导下，全国各族人民万众一心，克难攻坚，完成了全年经济社会发展主要目标任务，全面建成小康社会迈出坚实步伐，全面深化改革实现良好开局，全面依法治国开启新征程，全面从严治党取得新进展。

一年来，我国经济社会发展总体平稳，稳中有进。“稳”的主要标志是，经济运行处于合理区间。增速稳，国内生产总值达到63.6万亿元，比上年增长7.4%，在世界主要经济体中名列前茅。就业稳，城镇新增就业1322万人，高于上年。价格稳，居民消费价格上涨2%。“进”的总体特征是，发展的协调性和可持续性增强。经济结构有新的优化，粮食产量达到6.07亿吨，消费对经济增长的贡献率上升3个百分点，达到51.2%，服务业增加值比重由46.9%提高到48.2%，新产业、新业态、新商业模式不断涌现。中西部地区经济增速快于东部地区。发展质量有新的提升，一般公共预算收入增长8.6%，研究与试验发展经费支出与国内生产总值之比超过2%，能耗强度下降4.8%，是近年来最大降幅。人民生活有新的改善，全国居民人均可支配收入实际增长8%，快于经济增长；农村居民人均可支配收入实际增长9.2%，快于城镇居民收入增长；农村贫困人口减少1232万人；6600多万农村人口饮水安全问题得到解决；出境旅游超过1亿人次。改革开放有新的突破，全面深化改革系列重点任务启动实施，本届政府减少1/3行政审批事项的目标提前实现。这份成绩单的确来之不易，它凝聚着全国各族人民的心血和汗水，坚定了我们奋勇前行的决心和信心。

过去一年，困难和挑战比预想的大。我们迎难而上，主要做了以下工作。

一是在区间调控基础上实施定向调控，保持经济稳定增长。面对经济下行压力加大态势，我们保持战略定力，稳定宏观经济政策，没有采取短期强刺激措施，而是继续创新宏观调控思路和方式，实行定向调控，激活力、补短板、强实体。把握经济运行合理区间的上下限，抓住发展中的突出矛盾和结构性问题，定向施策，聚焦靶心，精准发力。向促改革要动力，向调结构要助力，向惠民生要潜力，既扩大市场需求，又增加有效供给，努力做到结构调优而不失速。

有效实施积极的财政政策和稳健的货币政策。实行定向减税和普遍性降费，拓宽小微企业税收优惠政策范围，扩大“营改增”试点。加快财政支出进度，积极盘活存量资金。灵活运用货币政策工具，采取定向降准、定向再贷款、非对称降息等措施，加大对经济社会发展薄弱环节的支持力度，小微企业、“三农”贷款增速比各项贷款平均增速分别高4.2和0.7个百分点。同时，完善金融监管，坚决守住不发生区域性系统性风险的底线。

二是深化改革开放，激发经济社会发展活力。针对束缚发展的体制机制障碍，我们通过全面深化改革，以释放市场活力对冲经济下行压力，啃了不少硬骨头，经济、政治、文化、社会、生态文明等体制改革全面推进。

扎实推动重点改革。制定并实施深化财税体制改革总体方案，预算管理制度和税制改革取得重要进展，专项转移支付项目比上年减少1/3以上，一般性转移支付比重增加，地方政府性债务管理得到加强。存款利率和汇率浮动区间扩大，民营银行试点迈出新步伐，“沪港通”试点启动，外汇储备、保险资金运用范围拓展。能源、交通、环保、通信等领域价格改革加快。启动科技资金管理、考试招生、户籍、机关事业单位养老保险制度等改革。

继续把简政放权、放管结合作为改革的重头戏。国务院各部门全年取消和下放246项行政审批事项，取消评比达标表彰项目29项、职业资格许可和认定事项149项，再次修订投资项目核准目录，大幅缩减核准范围。着力改革商事制度，新登记注册市场主体达到1293万户，其中新登记注册企业增长45.9%，形成新的创业热潮。经济增速放缓，新增就业不降反增，显示了改革的巨大威力和市场的无限潜力。

以开放促改革促发展。扩展上海自由贸易试验区范围，新设广东、天津、福建自由贸易试验区。稳定出口，增加进口，出口占国际市场份额继续提升。实际使用外商直接投资1196亿美元，居世界首位。对外直接投资1029亿美元，与利用外资并驾齐驱。中国与冰岛、瑞士自贸区启动实施，中韩、中澳自贸区完成实质性谈判。铁路、电力、油气、通信等领域对外合作取得重要成果，中国装备正大步走向世界。

三是加大结构调整力度，增强发展后劲。在结构性矛盾突出的情况下，我们积极作为，有扶有控，多办当前急需又利长远的事，夯实经济社会发展根基。

不断巩固农业基础。加大强农惠农富农政策力度，实现粮食产量“十一连增”、农民收入“五连快”。农业综合生产能力稳步提高，农业科技和机械化水平持续提升，重大水利工程建设进度加快，新增节水灌溉面积223万公顷，新建改建农村公路23万公里。新一轮退耕还林还草启动实施。农村土地确权登记颁证有序进行，农业新型经营主体加快成长。

大力调整产业结构。着力培育新的增长点，促进服务业加快发展，支持发展移动互联网、集成电路、高端装备制造、新能源汽车等战略性新兴产业，互联网金融异军突起，电子商务、物流快递等新业态快速成长，众多“创客”脱颖而出，文化创意产业蓬勃发展。同时，继续化解过剩产能，钢铁、水泥等15个重点行业淘汰落后产能年度任务如期完成。加强雾霾治理，淘汰黄标车和老旧车指标超额完成。

推进基础设施建设和区域协调发展。京津冀协同发展、长江经济带建设取得重要进展。新建铁路投产里程8427公里，高速铁路运营里程达1.6万公里，占世界的60%以上。高速公路通车里程达11.2万公里，水路、民航、管道建设进一步加强。农网改造稳步进行。宽带用户超过7.8亿户。经过多年努力，南水北调中线一期工程正式通水，惠及沿线亿万群众。

实施创新驱动发展战略。着力打通科技成果转化通道，扩大中关村国家自主创新示范区试点政策实施范围，推进科技资源开放共享，科技人员创新活力不断释放。超级计算、探月工程、卫星应用等重大科研项目取得新突破，我国自主研制的支线客机飞上蓝天。

四是织密织牢民生保障网，增进人民福祉。我们坚持以人为本，持续增加民生投入，保基本、兜底线、建机制，尽管财政收入增速放缓、支出压力加大，但财政用于民生的比例达到70%以上。

加强就业和社会保障。完善就业促进政策，推出创业引领计划，高校毕业生就业稳中有升。统一城乡居民基本养老保险制度，企业退休人员基本养老金水平又提高10%。新开工保障性安居工程740万套，基本建成511万套。全面建立临时救助制度，城乡低保标准分别提高9.97%和14.1%，残疾军人、烈属和老复员军人等优抚对象抚恤和生活补助标准提高20%以上。

继续促进教育公平。加强贫困地区义务教育薄弱学校建设，提高家庭经济困难学生资助水平，国家助学贷款资助标准大幅上调。中等职业学校免学费补助政策扩大到三年。实行义务教育免试就近入学政策，28个省份实现了农民工随迁子女在流入地参加高考。贫困地区农村学生上重点高校人数连续两年增长10%以上。经过努力，全国财政性教育经费支出占国内生产总值比例超过4%。

深入推进医药卫生改革发展。城乡居民大病保险试点扩大到所有省份，疾病应急救助制度基本建立，全民医保覆盖面超过95%。基层医疗卫生机构综合改革深化，县乡村服务网络逐步完善。公立医院改革试点县市达到1300多个。

积极发展文化事业和文化产业。推动重大文化惠民项目建设，广播电视“村村通”工程向“户户通”升级。实施文艺精品战略，完善现代文化市场体系。群众健身活动蓬勃开展，成功举办南京青奥会。

五是创新社会治理，促进和谐稳定。我们妥善应对自然灾害和突发事件，有序化解社会矛盾，建立健全机制，强化源头防范，保障人民生命安全，维护良好的社会秩序。

去年云南鲁甸、景谷等地发生较强地震，我们及时高效展开抗震救灾，灾后恢复重建顺利推进。积极援非抗击埃博拉疫情，有效防控疫情输入。加强安全生产工作，事故总量、重特大事故、重点行业事故持续下降。着力治理餐桌污染，食品药品安全形势总体稳定。

我们大力推进依法行政，国务院提请全国人大常委会制定修订食品安全法等法律15件，制定修订企业信息公示暂行条例等行政法规38件。政务公开深入推进，政府重大决策和政策以多种形式向社会广泛征求意见。完成第三次全国经济普查。改革信访工作制度。法律援助范围从低保群体扩大到低收入群体。加强城乡社区建设，行业协会商会等四类社会组织实现直接登记。严厉打击各类犯罪活动，强化社会治安综合治理，维护了国家安全和公共安全。

我们严格落实党中央八项规定精神，持之以恒纠正“四风”。严格执行国务院“约法三章”，政府性楼堂馆所、机关事业单位人员编制、“三公”经费得到有效控制。加大行政监察和审计监督力度，推进党风廉政建设和反腐败斗争，严肃查处违纪违法案件，一批腐败分子得到应有惩处。

我们狠抓重大政策措施的落实，认真开展督查，引入第三方评估和社会评价，建立长效机制，有力促进了各项工作。

一年来，外交工作成果丰硕。习近平主席等国家领导人出访多国，出席二十国集团领导人峰会、金砖国家领导人会晤、上海合作组织峰会、东亚合作领导人系列会议、亚欧首脑会议、达沃斯论坛等重大活动。成功举办亚太经合组织第二十二次领导人非正式会议、亚信会议第四次峰会、博鳌亚洲论坛。积极参与多边机制建立和国际规则制定。大国外交稳中有进，周边外交呈现新局面，同发展中国家合作取得新进展，经济外交成果显著。推进丝绸之路经济带和21世纪海上丝绸之路建设，筹建亚洲基础设施投资银行，设立丝路基金。我们与各国的交往合作越来越紧密，中国在国际舞台上负责任大国形象日益彰显。

各位代表！

一年来取得的成绩，是以习近平同志为核心的党中央统揽全局、科学决策的结果，是全党全军全国各族人民共同努力、不懈奋斗的结果。我代表国务院，向全国各族人民，向各民主党派、各人民团体和各界人士，表示诚挚感谢！向香港特别行政区同胞、澳门特别行政区同胞、台湾同胞和海外侨胞，表示诚挚感谢！向关心和支持中国现代化建设事业的各国政府、国际组织和各国朋友，表示诚挚感谢！

我们既要看到成绩，更要看到前进中的困难和挑战。投资增长乏力，新的消费热点不多，国际市场没有大的起色，稳增长难度加大，一些领域仍存在风险隐患。工业产品价格持续下降，生产要素成本上升，小微企业融资难融资贵问题突出，企业生产经营困难增多。经济发展方式比较粗放，创新能力不足，产能过剩问题突出，农业基础薄弱。群众对医疗、养老、住房、交通、教育、收入分配、食品安全、社会治安等还有不少不满意的地方。有些地方环境污染严重，重大安全事故时有发生。政府工作还存在不足，有些政策措施落实不到位。少数政府机关工作人员乱作为，一些腐败问题触目惊心，有的为官不为，在其位不谋其政，该办的事不办。我们要直面问题，安不忘危，治不忘乱，勇于担当，不辱历史使命，不负人民重托。

二、2015年工作总体部署

我国是世界上最大的发展中国家，仍处于并将长期处于社会主义初级阶段，发展是硬道理，是解决一切问题的基础和关键。化解各种矛盾和风险，跨越“中等收入陷阱”，实现现代化，根本要靠发展，发展必须有合理的增长速度。同时，我国经济发展进入新常态，正处在爬坡过坎的关口，体制机制弊端和结构性矛盾是“拦路虎”，不深化改革和调整经济结构，就难以实现平稳健康发展。我们必须毫不动摇坚持以经济建设为中心，切实抓好发展这个执政兴国第一要务。必须坚持不懈依靠改革推动科学发展，加快转变经济发展方式，实现有质量有效益可持续的发展。

当前，世界经济正处于深度调整之中，复苏动力不足，地缘政治影响加重，不确定因素增多，推动增长、增加就业、调整结构成为国际社会共识。我国经济下行压力还在加大，发展中深层次矛盾凸显，今年面临的困难可能比去年还要大。同时，我国发展仍处于可以大有作为的重要战略机遇期，有巨大的潜力、韧性和回旋余地。新型工业化、信息化、城镇化、农业现代化持续推进，发展基础日益雄厚，改革红利正在释放，宏观调控积累了丰富经验。我们必须增强忧患意识，坚定必胜信念，牢牢把握发展的主动权。

新的一年是全面深化改革的关键之年，是全面推进依法治国的开局之年，也是稳增长调结构的紧要之年。政府工作的总体要求是：高举中国特色社会主义伟大旗帜，以邓小平理论、“三个代表”重要思想、科学发展观为指导，全面贯彻党的十八大和十八届三中、四中全会精神，贯彻落实习近平总书记系列重要讲话精神，按照“四个全面”战略布局，主动适应和引领经济发展新常态，坚持稳中求进工作总基调，保持经济运行在合理区间，着力提高经济发展质量和效益，把转方式调结构放到更加重要位置，狠抓改革攻坚，突出创新驱动，强化风险防控，加强民生保障，处理好改革发展稳定关系，全面推进社会主义经济建设、政治建设、文化建设、社会建设、生态文明建设，促进经济平稳健康发展和社会和谐稳定。

我们要把握好总体要求，着眼于保持中高速增长和迈向中高端水平“双目标”，坚持稳政策稳预期和促改革调结构“双结合”，打造大众创业、万众创新和增加公共产品、公共服务“双引擎”，推动发展调速不减势、量增质更优，实现中国经济提质增效升级。

今年经济社会发展的主要预期目标是：国内生产总值增长7%左右，居民消费价格涨幅3%左右，城镇新增就业1000万人以上，城镇登记失业率4.5%以内，进出口增长6%左右，国际收支基本平衡，居民收入增长与经济发展同步。能耗强度下降3.1%以上，主要污染物排放继续减少。

经济增长预期7%左右，考虑了需要和可能，与全面建成小康社会目标相衔接，与经济总量扩大和结构升级的要求相适应，符合发展规律，符合客观实际。以这样的速度保持较长时期发展，实现现代化的物质基础就会更加雄厚。稳增长也是为了保就业，随着服务业比重上升、小微企业增多和经济体量增大，7%左右的速度可以实现比较充分的就业。各地要从实际出发，积极进取、挖掘潜力，努力争取更好结果。

做好今年政府工作，要把握好以下三点。

第一，稳定和完善宏观经济政策。继续实施积极的财政政策和稳健的货币政策，更加注重预调微调，更加注重定向调控，用好增量，盘活存量，重点支持薄弱环节。以微观活力支撑宏观稳定，以供给创新带动需求扩大，以结构调整促进总量平衡，确保经济运行在合理区间。

积极的财政政策要加力增效。今年拟安排财政赤字1.62万亿元，比去年增加2700亿元，赤字率从去年的2.1%提高到2.3%。其中，中央财政赤字1.12万亿元，增加1700亿元；地方财政赤字5000亿元，增加1000亿元。处理好债务管理与稳增长的关系，创新和完善地方政府举债融资机制。适当发行专项债券。保障符合条件的在建项目后续融资，防范和化解风险隐患。优化财政支出结构，大力盘活存量资金，提高使用效率。继续实行结构性减税和普遍性降费，进一步减轻企业特别是小微企业负担。

稳健的货币政策要松紧适度。广义货币M2预期增长12%左右，在实际执行中，根据经济发展需要，也可以略高些。加强和改善宏观审慎管理，灵活运用公开市场操作、利率、存款准备金率、再贷款等货币政策工具，保持货币信贷和社会融资规模平稳增长。加快资金周转，优化信贷结构，提高直接融资比重，降低社会融资成本，让更多的金融活水流向实体经济。

第二，保持稳增长与调结构的平衡。我国发展面临“三期叠加”矛盾，资源环境约束加大，劳动力等要素成本上升，高投入、高消耗、偏重数量扩张的发展方式已经难以为继，必须推动经济在稳定增长中优化结构。既要稳住速度，确保经济平稳运行，确保居民就业和收入持续增加，为调结构转方式创造有利条件；又要调整结构，夯实稳增长的基础。要增加研发投入，提高全要素生产率，加强质量、标准和品牌建设，促进服务业和战略性新兴产业比重提高、水平提升，优化经济发展空间格局，加快培育新的增长点和增长极，实现在发展中升级、在升级中发展。

第三，培育和催生经济社会发展新动力。当前经济增长的传统动力减弱，必须加大结构性改革力度，加快实施创新驱动发展战略，改造传统引擎，打造新引擎。一方面，增加公共产品和服务供给，加大政府对教育、卫生等的投入，鼓励社会参与，提高供给效率。这既能补短板、惠民生，也有利于扩需求、促发展。另一方面，推动大众创业、万众创新。这既可以扩大就业、增加居民收入，又有利于促进社会纵向流动和公平正义。我国有13亿人口、9亿劳动力资源，人民勤劳而智慧，蕴藏着无穷的创造力，千千万万个市场细胞活跃起来，必将汇聚成发展的巨大动能，一定能够顶住经济下行压力，让中国经济始终充满勃勃生机。政府要勇于自我革命，给市场和社会留足空间，为公平竞争搭好舞台。个人和企业要勇于创业创新，全社会要厚植创业创新文化，让人们在创造财富的过程中，更好地实现精神追求和自身价值。

今年是“十二五”收官之年，我们要在完成“十二五”经济社会发展目标任务的同时，以改革的精神、创新的理念和科学的方法，做好“十三五”规划纲要编制工作，谋划好未来五年的发展蓝图。

三、把改革开放扎实推向纵深

改革开放是推动发展的制胜法宝。必须以经济体制改革为重点全面深化改革，统筹兼顾，真抓实干，在牵动全局的改革上取得新突破，增强发展新动能。

加大简政放权、放管结合改革力度。今年再取消和下放一批行政审批事项，全部取消非行政许可审批，建立规范行政审批的管理制度。深化商事制度改革，进一步简化注册资本登记，逐步实现“三证合一”，清理规范中介服务。制定市场准入负面清单，公布省级政府权力清单、责任清单，切实做到法无授权不可为、法定职责必须为。地方政府对应当放给市场和社会的权力，要彻底放、不截留，对上级下放的审批事项，要接得住、管得好。加强事中事后监管，健全为企业和社会服务一张网，推进社会信用体系建设，建立全国统一的社会信用代码制度和信用信息共享交换平台，依法保护企业和个人信息安全。大道至简，有权不可任性。各级政府都要建立简政放权、转变职能的有力推进机制，给企业松绑，为创业提供便利，营造公平竞争环境。所有行政审批事项都要简化程序，明确时限，用政府权力的“减法”，换取市场活力的“乘法”。

多管齐下改革投融资体制。大幅缩减政府核准投资项目范围，下放核准权限。大幅减少投资项目前置审批，实行项目核准网上并联办理。大幅放宽民间投资市场准入，鼓励社会资本发起设立股权投资基金。政府采取投资补助、资本金注入、设立基金等办法，引导社会资本投入重点项目。以用好铁路发展基金为抓手，深化铁路投融资改革。在基础设施、公用事业等领域，积极推广政府和社会资本合作模式。

不失时机加快价格改革。改革方向是发挥市场在资源配置中的决定性作用，大幅缩减政府定价种类和项目，具备竞争条件的商品和服务价格原则上都要放开。取消绝大部分药品政府定价，下放一批基本公共服务收费定价权。扩大输配电价改革试点，推进农业水价改革，健全节能环保价格政策。完善资源性产品价格，全面实行居民阶梯价格制度。同时必须加强价格监管，规范市场秩序，确保低收入群众基本生活。

推动财税体制改革取得新进展。实行全面规范、公开透明的预算管理制度，除法定涉密信息外，中央和地方所有部门预决算都要公开，全面接受社会监督。提高国有资本经营预算调入一般公共预算的比例。推行中期财政规划管理。制定盘活财政存量资金的有效办法。力争全面完成“营改增”，调整完善消费税政策，扩大资源税从价计征范围。提请修订税收征管法。改革转移支付制度，完善中央和地方的事权与支出责任，合理调整中央和地方收入划分。

围绕服务实体经济推进金融改革。推动具备条件的民间资本依法发起设立中小型银行等金融机构，成熟一家，批准一家，不设限额。深化农村信用社改革，稳定其县域法人地位。发挥好开发性金融、政策性金融在增加公共产品供给中的作用。推出存款保险制度。推进利率市场化改革，健全中央银行利率调控框架。保持人民币汇率处于合理均衡水平，增强人民币汇率双向浮动弹性。稳步实现人民币资本项目可兑换，扩大人民币国际使用，加快建设人民币跨境支付系统，完善人民币全球清算服务体系，开展个人投资者境外投资试点，适时启动“深港通”试点。加强多层次资本市场体系建设，实施股票发行注册制改革，发展服务中小企业的区域性股权市场，开展股权众筹融资试点，推进信贷资产证券化，扩大企业债券发行规模，发展金融衍生品市场。推出巨灾保险、个人税收递延型商业养老保险。创新金融监管，防范和化解金融风险。大力发展普惠金融，让所有市场主体都能分享金融服务的雨露甘霖。

深化国企国资改革。准确界定不同国有企业功能，分类推进改革。加快国有资本投资公司、运营公司试点，打造市场化运作平台，提高国有资本运营效率。有序实施国有企业混合所有制改革，鼓励和规范投资项目引入非国有资本参股。加快电力、油气等体制改革。多渠道解决企业办社会负担和历史遗留问题，保障职工合法权益。完善现代企业制度，改革和健全企业经营者激励约束机制。要加强国有资产监管，防止国有资产流失，切实提高国有企业的经营效益。

非公有制经济是我国经济的重要组成部分。必须毫不动摇鼓励、支持、引导非公有制经济发展，注重发挥企业家才能，全面落实促进民营经济发展的政策措施，增强各类所有制经济活力，让各类企业法人财产权依法得到保护。

继续推进科技、教育、文化、医药卫生、养老保险、事业单位、住房公积金等领域改革。发展需要改革添动力，群众期盼改革出实效，我们要努力交出一份为发展加力、让人民受益的改革答卷。

开放也是改革。必须实施新一轮高水平对外开放，加快构建开放型经济新体制，以开放的主动赢得发展的主动、国际竞争的主动。

推动外贸转型升级。完善出口退税负担机制，自2015年起增量部分由中央财政全额负担，让地方和企业吃上“定心丸”。清理规范进出口环节收费，建立并公开收费项目清单。实施培育外贸竞争新优势的政策措施，促进加工贸易转型，发展外贸综合服务平台和市场采购贸易，扩大跨境电子商务综合试点，增加服务外包示范城市数量，提高服务贸易比重。实施更加积极的进口政策，扩大先进技术、关键设备、重要零部件等进口。

更加积极有效利用外资。修订外商投资产业指导目录，重点扩大服务业和一般制造业开放，把外商投资限制类条目缩减一半。全面推行普遍备案、有限核准的管理制度，大幅下放鼓励类项目核准权，积极探索准入前国民待遇加负面清单管理模式。修订外商投资相关法律，健全外商投资监管体系，打造稳定公平透明可预期的营商环境。

加快实施走出去战略。鼓励企业参与境外基础设施建设和产能合作，推动铁路、电力、通信、工程机械以及汽车、飞机、电子等中国装备走向世界，促进冶金、建材等产业对外投资。实行以备案制为主的对外投资管理方式。扩大出口信用保险规模，对大型成套设备出口融资应保尽保。拓宽外汇储备运用渠道，健全金融、信息、法律、领事保护服务。注重风险防范，提高海外权益保障能力。让中国企业走得出、走得稳，在国际竞争中强筋健骨、发展壮大。

构建全方位对外开放新格局。推进丝绸之路经济带和21世纪海上丝绸之路合作建设。加快互联互通、大通关和国际物流大通道建设。构建中巴、孟中印缅等经济走廊。扩大内陆和沿边开放，促进经济技术开发区创新发展，提高边境经济合作区、跨境经济合作区发展水平。积极推动上海和广东、天津、福建自贸试验区建设，在全国推广成熟经验，形成各具特色的改革开放高地。

统筹多双边和区域开放合作。维护多边贸易体制，推动信息技术协定扩围，积极参与环境产品、政府采购等国际谈判。加快实施自贸区战略，尽早签署中韩、中澳自贸协定，加快中日韩自贸区谈判，推动与海合会、以色列等自贸区谈判，力争完成中国－东盟自贸区升级谈判和区域全面经济伙伴关系协定谈判，建设亚太自贸区。推进中美、中欧投资协定谈判。中国是负责任、敢担当的国家，我们愿做互利共赢发展理念的践行者、全球经济体系的建设者、经济全球化的推动者。

四、协调推动经济稳定增长和结构优化

稳增长和调结构相辅相成。我们既要全力保持经济在合理区间运行，又要积极促进经济转型升级、行稳致远。

加快培育消费增长点。鼓励大众消费，控制“三公”消费。促进养老家政健康消费，壮大信息消费，提升旅游休闲消费，推动绿色消费，稳定住房消费，扩大教育文化体育消费。全面推进“三网”融合，加快建设光纤网络，大幅提升宽带网络速率，发展物流快递，把以互联网为载体、线上线下互动的新兴消费搞得红红火火。建立健全消费品质量安全监管、追溯、召回制度，严肃查处制售假冒伪劣行为，保护消费者合法权益。扩大消费要汇小溪成大河，让亿万群众的消费潜力成为拉动经济增长的强劲动力。

增加公共产品有效投资。确保完成“十二五”规划重点建设任务，启动实施一批新的重大工程项目。主要是：棚户区和危房改造、城市地下管网等民生项目，中西部铁路和公路、内河航道等重大交通项目，水利、高标准农田等农业项目，信息、电力、油气等重大网络项目，清洁能源及油气矿产资源保障项目，传统产业技术改造等项目，节能环保和生态建设项目。今年中央预算内投资增加到4776亿元，但政府不唱“独角戏”，要更大激发民间投资活力，引导社会资本投向更多领域。铁路投资要保持在8000亿元以上，新投产里程8000公里以上，在全国基本实现高速公路电子不停车收费联网，使交通真正成为发展的先行官。重大水利工程已开工的57个项目要加快建设，今年再开工27个项目，在建重大水利工程投资规模超过8000亿元。棚改、铁路、水利等投资多箭齐发，重点向中西部地区倾斜，使巨大的内需得到更多释放。

加快推进农业现代化。坚持“三农”重中之重地位不动摇，加快转变农业发展方式，让农业更强、农民更富、农村更美。

今年粮食产量要稳定在5.5亿吨以上，保障粮食安全和主要农产品供给。坚守耕地红线，全面开展永久基本农田划定工作，实施耕地质量保护与提升行动，推进土地整治，增加深松土地1333万公顷。加强农田水利基本建设，大力发展节水农业。加快新技术、新品种、新农机研发推广应用。引导农民瞄准市场调整种养结构，支持农产品加工特别是主产区粮食就地转化，开展粮食作物改为饲料作物试点。综合治理农药兽药残留等问题，全面提高农产品质量和食品安全水平。

新农村建设要惠及广大农民。突出加强水和路的建设，今年再解决6000万农村人口饮水安全问题，新建改建农村公路20万公里，全面完成西部边远山区溜索改桥任务。力争让最后20多万无电人口都能用上电。以垃圾、污水为重点加强环境治理，建设美丽宜居乡村。多渠道促进农民增收，保持城乡居民收入差距缩小势头。持续打好扶贫攻坚战，深入推进集中连片特困地区扶贫开发，实施精准扶贫、精准脱贫。难度再大，今年也要再减少农村贫困人口1000万人以上。

推进农业现代化，改革是关键。要在稳定家庭经营的基础上，支持种养大户、家庭农牧场、农民合作社、产业化龙头企业等新型经营主体发展，培养新型职业农民，推进多种形式适度规模经营。做好土地确权登记颁证工作，审慎开展农村土地征收、集体经营性建设用地入市、宅基地制度、集体产权制度等改革试点。在改革中，要确保耕地数量不减少、质量不下降、农民利益有保障。深化供销社、农垦、种业、国有林场林区等改革，办好农村改革试验区和现代农业示范区。完善粮食最低收购价和临时收储政策，改进农产品目标价格补贴办法。加强涉农资金统筹整合和管理。无论财政多困难，惠农政策只能加强不能削弱，支农资金只能增加不能减少。

推进新型城镇化取得新突破。城镇化是解决城乡差距的根本途径，也是最大的内需所在。要坚持以人为核心，以解决三个1亿人问题为着力点，发挥好城镇化对现代化的支撑作用。

加大城镇棚户区和城乡危房改造力度。今年保障性安居工程新安排740万套，其中棚户区改造580万套，增加110万套，把城市危房改造纳入棚改政策范围。农村危房改造366万户，增加100万户，统筹推进农房抗震改造。住房保障逐步实行实物保障与货币补贴并举，把一些存量房转为公租房和安置房。对居住特别困难的低保家庭，给予住房救助。坚持分类指导，因地施策，落实地方政府主体责任，支持居民自住和改善性住房需求，促进房地产市场平稳健康发展。

用改革的办法解决城镇化难点问题。抓紧实施户籍制度改革，落实放宽户口迁移政策。对已在城镇就业和居住但尚未落户的外来人口，以居住证为载体提供相应基本公共服务，取消居住证收费。建立财政转移支付与市民化挂钩机制，合理分担农民工市民化成本。建立规范多元可持续的城市建设投融资机制。坚持节约集约用地，稳妥建立城乡统一的建设用地市场，完善和拓展城乡建设用地增减挂钩试点。加强资金和政策支持，扩大新型城镇化综合试点。

提升城镇规划建设管理水平。制定实施城市群规划，有序推进基础设施和基本公共服务同城化。完善设市标准，实行特大镇扩权增能试点，控制超大城市人口规模，提升地级市、县城和中心镇产业和人口承载能力，方便农民就近城镇化。发展智慧城市，保护和传承历史、地域文化。加强城市供水供气供电、公交和防洪防涝设施等建设。坚决治理污染、拥堵等城市病，让出行更方便、环境更宜居。

拓展区域发展新空间。统筹实施“四大板块”和“三个支撑带”战略组合。在西部地区开工建设一批综合交通、能源、水利、生态、民生等重大项目，落实好全面振兴东北地区等老工业基地政策措施，加快中部地区综合交通枢纽和网络等建设，支持东部地区率先发展，加大对革命老区、民族地区、边疆地区、贫困地区支持力度，完善差别化的区域发展政策。把“一带一路”建设与区域开发开放结合起来，加强新亚欧大陆桥、陆海口岸支点建设。推进京津冀协同发展，在交通一体化、生态环保、产业升级转移等方面率先取得实质性突破。推进长江经济带建设，有序开工黄金水道治理、沿江码头口岸等重大项目，构筑综合立体大通道，建设产业转移示范区，引导产业由东向西梯度转移。加速资源枯竭型城市转型升级。加强中西部重点开发区建设，深化泛珠三角等区域合作。 我国是海洋大国，要编制实施海洋战略规划，发展海洋经济，保护海洋生态环境，提高海洋科技水平，强化海洋综合管理，加强海上力量建设，坚决维护国家海洋权益，妥善处理海上纠纷，积极拓展双边和多边海洋合作，向海洋强国的目标迈进。

推动产业结构迈向中高端。制造业是我们的优势产业。要实施“中国制造2025”，坚持创新驱动、智能转型、强化基础、绿色发展，加快从制造大国转向制造强国。采取财政贴息、加速折旧等措施，推动传统产业技术改造。坚持有保有压，化解过剩产能，支持企业兼并重组，在市场竞争中优胜劣汰。促进工业化和信息化深度融合，开发利用网络化、数字化、智能化等技术，着力在一些关键领域抢占先机、取得突破。

新兴产业和新兴业态是竞争高地。要实施高端装备、信息网络、集成电路、新能源、新材料、生物医药、航空发动机、燃气轮机等重大项目，把一批新兴产业培育成主导产业。制定“互联网+”行动计划，推动移动互联网、云计算、大数据、物联网等与现代制造业结合，促进电子商务、工业互联网和互联网金融健康发展，引导互联网企业拓展国际市场。国家已设立400亿元新兴产业创业投资引导基金，要整合筹措更多资金，为产业创新加油助力。

服务业就业容量大，发展前景广。要深化服务业改革开放，落实财税、土地、价格等支持政策以及带薪休假等制度，大力发展旅游、健康、养老、创意设计等生活和生产服务业。深化流通体制改革，加强大型农产品批发、仓储和冷链等现代物流设施建设，努力大幅降低流通成本。

以体制创新推动科技创新。创新创造关键在人。要加快科技成果使用处置和收益管理改革，扩大股权和分红激励政策实施范围，完善科技成果转化、职务发明法律制度，使创新人才分享成果收益。制定促进科研人员流动政策，改革科技评价、职称评定和国家奖励制度，推进科研院所分类改革。引进国外高质量人才和智力。深入实施知识产权战略行动计划，依法打击侵权行为，切实保护发明创造，让创新之树枝繁叶茂。

企业是技术创新的主体。要落实和完善企业研发费用加计扣除、高新技术企业扶持等普惠性政策，鼓励企业增加创新投入。支持企业更多参与重大科技项目实施、科研平台建设，推进企业主导的产学研协同创新。大力发展众创空间，增设国家自主创新示范区，办好国家高新区，发挥集聚创新要素的领头羊作用。中小微企业大有可为，要扶上马、送一程，使“草根”创新蔚然成风、遍地开花。

提高创新效率重在优化科技资源配置。要改革中央财政科技计划管理方式，建立公开统一的国家科技管理平台。政府重点支持基础研究、前沿技术和重大关键共性技术研究，鼓励原始创新，加快实施国家科技重大项目，向社会全面开放重大科研基础设施和大型科研仪器。把亿万人民的聪明才智调动起来，就一定能够迎来万众创新的浪潮。

五、持续推进民生改善和社会建设

立国之道，惟在富民。要以增进民生福祉为目的，加快发展社会事业，改革完善收入分配制度，千方百计增加居民收入，促进社会公平正义与和谐进步。

着力促进创业就业。坚持就业优先，以创业带动就业。今年高校毕业生749万人，为历史最高。要加强就业指导和创业教育，落实高校毕业生就业促进计划，鼓励到基层就业。实施好大学生创业引领计划，支持到新兴产业创业。做好结构调整、过剩产能化解中失业人员的再就业工作。统筹农村转移劳动力、城镇困难人员、退役军人就业，实施农民工职业技能提升计划，落实和完善失业保险支持企业稳定就业岗位政策。全面治理拖欠农民工工资问题，健全劳动监察和争议处理机制，让法律成为劳动者权益的守护神。

加强社会保障和增加居民收入。企业退休人员基本养老金标准提高10%。城乡居民基础养老金标准统一由55元提高到70元。推进城镇职工基础养老金全国统筹。降低失业保险、工伤保险等缴费率。完善最低工资标准调整机制。落实机关事业单位养老保险制度改革措施，同步完善工资制度，对基层工作人员给予政策倾斜。在县以下机关建立公务员职务和职级并行制度。加强重特大疾病医疗救助，全面实施临时救助制度，让遇到急难特困的群众求助有门、受助及时。对困境儿童、高龄和失能老人、重度和贫困残疾人等特困群体，健全福利保障制度和服务体系。继续提高城乡低保水平，提升优抚对象抚恤和生活补助标准。提高工资和保障标准等政策的受益面广，各级政府一定要落实到位。民之疾苦，国之要事，我们要竭尽全力，坚决把民生底线兜住兜牢。

促进教育公平发展和质量提升。教育是今天的事业、明天的希望。要坚持立德树人，增强学生的社会责任感、创新精神、实践能力，培养中国特色社会主义建设者和接班人。深化省级政府教育统筹改革、高等院校综合改革和考试招生制度改革。加快义务教育学校标准化建设，改善薄弱学校和寄宿制学校基本办学条件。落实农民工随迁子女在流入地接受义务教育政策，完善后续升学政策。全面推进现代职业教育体系建设。引导部分地方本科高校向应用型转变，通过对口支援等方式支持中西部高等教育发展，继续提高中西部地区和人口大省高考录取率。建设世界一流大学和一流学科。加强特殊教育、学前教育、继续教育和民族地区各类教育。促进民办教育健康发展。加强教师队伍建设。为切实把教育事业办好，我们要保证投入，花好每一分钱。要畅通农村和贫困地区学子纵向流动的渠道，让每个人都有机会通过教育改变自身命运。

加快健全基本医疗卫生制度。完善城乡居民基本医保，财政补助标准由每人每年320元提高到380元，基本实现居民医疗费用省内直接结算，稳步推行退休人员医疗费用跨省直接结算。全面实施城乡居民大病保险制度。深化基层医疗卫生机构综合改革，加强全科医生制度建设，完善分级诊疗体系。全面推开县级公立医院综合改革，在100个地级以上城市进行公立医院改革试点，破除以药补医，降低虚高药价，合理调整医疗服务价格，通过医保支付等方式平衡费用，努力减轻群众负担。鼓励医生到基层多点执业，发展社会办医。开展省级深化医改综合试点。加快建立医疗纠纷预防调解机制。人均基本公共卫生服务经费补助标准由35元提高到40元，增量全部用于支付村医的基本公共卫生服务，方便几亿农民就地就近看病就医。加强重大疾病防控。积极发展中医药和民族医药事业。推进计划生育服务管理改革。健康是群众的基本需求，我们要不断提高医疗卫生水平，打造健康中国。

让人民群众享有更多更好文化发展成果。文化是民族的精神命脉和创造源泉。要践行社会主义核心价值观，弘扬中华优秀传统文化。繁荣发展哲学社会科学，发展文学艺术、新闻出版、广播影视、档案等事业，重视文物、非物质文化遗产保护。提供更多优秀文艺作品，倡导全民阅读，建设学习型社会，提高国民素质。深化文化体制改革，逐步推进基本公共文化服务标准化均等化，扩大公共文化设施免费开放范围，发挥基层综合性文化服务中心作用，促进传统媒体与新兴媒体融合发展。拓展中外人文交流，加强国际传播能力建设。发展全民健身、竞技体育和体育产业，做好2022年冬奥会申办工作。

加强和创新社会治理。深化社会组织管理制度改革，加快行业协会商会与行政机关脱钩。支持群团组织依法参与社会治理，发展专业社会工作、志愿服务和慈善事业。鼓励社会力量兴办养老设施，发展社区和居家养老。为农村留守儿童、妇女、老人提供关爱服务，建立未成年人社会保护制度，切实保障妇女儿童权益。提高公共突发事件防范处置和防灾救灾减灾能力。做好地震、气象、测绘、地质等工作。深入开展法治宣传教育，加强人民调解工作，完善法律援助制度，落实重大决策社会稳定风险评估机制，有效预防和化解社会矛盾。把信访纳入法治轨道，及时就地解决群众合理诉求。深化平安中国建设，健全立体化社会治安防控体系，依法惩治暴恐、黄赌毒、邪教、走私等违法犯罪行为，发展和规范网络空间，确保国家安全和公共安全。人的生命最为宝贵，要采取更坚决措施，全方位强化安全生产，全过程保障食品药品安全。

打好节能减排和环境治理攻坚战。环境污染是民生之患、民心之痛，要铁腕治理。今年，二氧化碳排放强度要降低3.1%以上，化学需氧量、氨氮排放都要减少2%左右，二氧化硫、氮氧化物排放要分别减少3%左右和5%左右。深入实施大气污染防治行动计划，实行区域联防联控，加强煤炭清洁高效利用，推动燃煤电厂超低排放改造，促进重点区域煤炭消费零增长。推广新能源汽车，治理机动车尾气，提高油品标准和质量，在重点区域内重点城市全面供应国五标准车用汽柴油。2005年底前注册营运的黄标车今年要全部淘汰。积极应对气候变化，扩大碳排放权交易试点。实施水污染防治行动计划，加强江河湖海水污染、水污染源和农业面源污染治理，实行从水源地到水龙头全过程监管。加强土壤污染防治。推行环境污染第三方治理。做好环保税立法工作。我们一定要严格环境执法，对偷排偷放者出重拳，让其付出沉重的代价；对姑息纵容者严问责，使其受到应有的处罚。

能源生产和消费革命，关乎发展与民生。要大力发展风电、光伏发电、生物质能，积极发展水电，安全发展核电，开发利用页岩气、煤层气。控制能源消费总量，加强工业、交通、建筑等重点领域节能。积极发展循环经济，大力推进工业废物和生活垃圾资源化利用。我国节能环保市场潜力巨大，要把节能环保产业打造成新兴的支柱产业。

森林草原、江河湿地是大自然赐予人类的绿色财富，必须倍加珍惜。要推进重大生态工程建设，拓展重点生态功能区，办好生态文明先行示范区，开展国土江河综合整治试点，扩大流域上下游横向补偿机制试点，保护好三江源。扩大天然林保护范围，有序停止天然林商业性采伐。今年新增退耕还林还草66.7万公顷，造林600万公顷。生态环保贵在行动、成在坚持，我们必须紧抓不松劲，一定要实现蓝天常在、绿水长流、永续发展。

六、切实加强政府自身建设

我们要全面推进依法治国，加快建设法治政府、创新政府、廉洁政府和服务型政府，增强政府执行力和公信力，促进国家治理体系和治理能力现代化。

坚持依宪施政，依法行政，把政府工作全面纳入法治轨道。宪法是我们根本的活动准则，各级政府及其工作人员都必须严格遵守。要尊法学法守法用法，依法全面履行职责，所有行政行为都要于法有据，任何政府部门都不得法外设权。深化行政执法体制改革，严格规范公正文明执法，加快推进综合执法，全面落实行政执法责任制。一切违法违规的行为都要追究，一切执法不公正不文明的现象都必须纠正。

坚持创新管理，强化服务，着力提高政府效能。提供基本公共服务尽可能采用购买服务方式，第三方可提供的事务性管理服务交给市场或社会去办。扎实开展政府协商，积极推进决策科学化民主化，重视发挥智库作用。全面实行政务公开，推广电子政务和网上办事。各级政府要自觉接受同级人大及其常委会的监督，接受人民政协的民主监督，认真听取人大代表、政协委员、民主党派、工商联、无党派人士和各人民团体的意见。我们的所有工作都要全面接受人民的监督，充分体现人民的意愿。

坚持依法用权，倡俭治奢，深入推进党风廉政建设和反腐败工作。认真落实党中央八项规定精神，坚持不懈纠正“四风”，继续严格执行国务院“约法三章”。腐败现象的一个共同特征就是权力寻租，要以权力瘦身为廉政强身，紧紧扎住制度围栏，坚决打掉寻租空间，努力铲除腐败土壤。加强行政监察，发挥审计监督作用，对公共资金、公共资源、国有资产严加监管。始终保持反腐高压态势，对腐败分子零容忍、严查处。对腐败行为，无论出现在领导机关，还是发生在群众身边，都必须严加惩治。

坚持主动作为，狠抓落实，切实做到勤政为民。经济发展进入新常态，精神面貌要有新状态。广大公务员特别是领导干部要始终把为人民谋发展增福祉作为最大责任，始终把现代化建设使命扛在肩上，始终把群众冷暖忧乐放在心头。各级政府要切实履行职责，狠抓贯彻落实，创造性开展工作。完善政绩考核评价机制，对实绩突出的要大力褒奖，对工作不力的要约谈诫勉，对为官不为、懒政怠政的要公开曝光、坚决追究责任。

各位代表！

我国是统一的多民族国家，巩固和发展平等团结互助和谐的社会主义民族关系，是全国各族人民的根本利益和共同责任。要坚持和完善民族区域自治制度，加大对欠发达的民族地区支持力度，扶持人口较少民族发展，推进兴边富民行动，保护和发展少数民族优秀传统文化及特色村镇，促进各民族交往交流交融。组织好西藏自治区成立50周年和新疆维吾尔自治区成立60周年庆祝活动。各族人民和睦相处、和衷共济、和谐发展，中华民族大家庭一定会更加繁荣昌盛、幸福安康。

我们要全面贯彻党的宗教工作基本方针，促进宗教关系和谐，维护宗教界合法权益，发挥宗教界人士和信教群众在促进经济社会发展中的积极作用。

我们要更好发挥海外侨胞和归侨侨眷参与祖国现代化建设、促进祖国和平统一、推进中外交流合作的独特作用，使海内外中华儿女的向心力不断增强。

各位代表！

建设巩固的国防和强大的军队，是维护国家主权、安全和发展利益的根本保障。要紧紧围绕党在新形势下的强军目标，坚持党对军队绝对领导的根本原则，统筹抓好各方面各领域军事斗争准备，保持边防海防空防安全稳定。全面加强现代后勤建设，加大国防科研和高新技术武器装备建设力度，发展国防科技工业。深化国防和军队改革，提高国防和军队建设法治化水平。加强现代化武装警察力量建设。增强全民国防意识，推进国防动员和后备力量建设。坚持国防建设和经济建设协调发展，促进军民融合深度发展。各级政府要始终如一地关心和支持国防和军队建设，坚定不移地巩固和促进军政军民团结。

各位代表！

我们将坚定不移地贯彻“一国两制”、“港人治港”、“澳人治澳”、高度自治方针，严格依照宪法和基本法办事。全力支持香港、澳门特别行政区行政长官和政府依法施政，发展经济，改善民生，推进民主，促进和谐。加强内地与港澳各领域交流合作，继续发挥香港、澳门在国家改革开放和现代化建设中的特殊作用。我们坚信，有中央政府一以贯之的大力支持，不断提升港澳自身竞争力，香港、澳门就一定能够保持长期繁荣稳定。

我们将坚持对台工作大政方针，巩固两岸坚持“九二共识”、反对“台独”的政治基础，保持两岸关系和平发展正确方向。努力推进两岸协商对话，推动经济互利融合，加强基层和青少年交流。依法保护台湾同胞权益，让更多民众分享两岸关系和平发展成果。我们期待两岸同胞不断增进了解互信，密切骨肉亲情，拉近心理距离，为实现祖国和平统一贡献力量。我们坚信，两岸关系和平发展是不可阻挡、不可逆转的历史潮流。

各位代表！

我们将继续高举和平发展合作共赢旗帜，统筹国内国际两个大局，始终不渝走和平发展道路，始终不渝奉行互利共赢开放战略，坚决维护国家主权安全发展利益，维护我国公民和法人海外合法权益，推动建立以合作共赢为核心的新型国际关系。深化与各大国战略对话和务实合作，构建健康稳定的大国关系框架。全面推进周边外交，打造周边命运共同体。加强同发展中国家团结合作，维护共同利益。积极参与国际多边事务，推动国际体系和秩序朝着更加公正合理方向发展。办好纪念中国人民抗日战争暨世界反法西斯战争胜利70周年相关活动，同国际社会共同维护二战胜利成果和国际公平正义。我们愿与世界各国携手并肩，维护更加持久的和平，建设更加繁荣的世界。

各位代表！

时代赋予中国发展兴盛的历史机遇。让我们紧密团结在以习近平同志为核心的党中央周围，高举中国特色社会主义伟大旗帜，凝神聚力，开拓创新，努力完成今年经济社会发展目标任务，为实现“两个一百年”奋斗目标、建成富强民主文明和谐的社会主义现代化国家、实现中华民族伟大复兴的中国梦作出新的更大贡献！
